# Fresh street: the development and feasibility of a place-based, subsidy for fresh fruit and vegetables

**DOI:** 10.1093/pubmed/fdaa190

**Published:** 2020-11-09

**Authors:** C Relton, M Crowder, M Blake, M Strong

**Affiliations:** Institute of Population Health Sciences, Queen Mary University of London, London E1 2AB, UK; School of Health and Related Research, University of Sheffield, Sheffield S1 4DA, UK; School of Health and Related Research, University of Sheffield, Sheffield S1 4DA, UK; Department of Geography, University of Sheffield, Sheffield, UK; School of Health and Related Research, University of Sheffield, Sheffield S1 4DA, UK

**Keywords:** food and nutrition, food environment, places

## Abstract

**Background:**

Many UK communities experience food insecurity, and consume diets high in energy-dense, nutrient poor, processed foods and low in fruit and vegetables (FV). We explored a novel area-based approach to promote FV consumption and healthy eating in one such community.

**Methods:**

We developed a weekly subsidy scheme for fresh FV with key local stakeholders in an area of socioeconomic deprivation in Northern England. The scheme (Fresh Street) offered five £1 vouchers to every household, regardless of income or household type. Vouchers were redeemable with local suppliers of fresh FV (not supermarkets). The feasibility of the scheme was assessed in four streets using rapid ethnographic assessment and voucher redemption information.

**Results:**

Local councillors and public health teams were supportive of the scheme. Most eligible households joined the scheme (*n* = 80/97, 83%), and 89.3% (17 849/19 982) of vouchers issued were redeemed. Householders reported that the scheme made them think about what they were eating, and prompted them to buy and eat more FV.

**Conclusions:**

This feasibility study reported high levels of acceptance for a place-based, household-level weekly FV subsidy scheme. Further research is required to evaluate the effectiveness of this approach to creating healthy diets, eating behaviours and food systems.

## Introduction

Many communities in the UK cannot afford food to make up a healthy diet.^1^^,^^2^^,^^3^^,^^5^ These communities consume diets low in fruit and vegetables (FV) and high in energy-dense, nutrient-poor processed foods. Those on low incomes are more likely to have a higher intake of sugar and saturated fatty acids, and lower intake of FV and dietary fibre than recommended.^4^ This leads to an increase in all-cause mortality and cardiovascular mortality.^6^

Price discounts applied at a population level have been associated with a positive shift in population level purchases of FV, with the effect persisting after removal of the discount.^7^ Targeted benefits such as vouchers and subsidies can be effective in increasing purchases of FV and improving the nutritional composition of food bought. There is mounting evidence (mostly from USA) that price discounts are effective in increasing purchasing and consumption^8^^,^^9^ of healthier foods. However, the majority of programmes target individual families—mainly low-income women, infant and children. In the USA, these programmes report significant increases in the intake of FV^10^ and savings in food expenditure.^11^

In the UK, the Department of Health & Social Care ‘Healthy Start’ programme provides vouchers worth £3.10 per week for FV, milk and infant formula^12^. This programme also targets individual low-income pregnant women and mothers with children under four in households on income support. Application to this programme is via healthcare providers, and vouchers are sent monthly. Although this programme increased spending on FV,^13^ uptake is rapidly declining with less than half of those eligible in the programme.^14^ This is in part due to the stigma associated with the targeted nature of the scheme.^15^

The purpose of this early phase study was to develop and test the feasibility and acceptability of a voucher scheme that targeted areas rather than individual families. The voucher scheme aimed to: (i) increase fresh FV consumption, (ii) encourage new purchasing, food preparation and eating patterns in the short term and (iii) reduce food poverty and improve health outcomes in the longer term in the UK.

## Methods

### Study design and setting

We developed an area-based voucher scheme for communities with high deprivation levels and low FV consumption.

The setting for this early phase development and feasibility study was situated in Barnsley, a town in the north of England with a population of ~240 000. Barnsley has many areas of high deprivation, poor health and poor diet. Almost 98% of this population is white, and 97% were born in the UK. Barnsley Metropolitan Borough Council identified low fresh FV intake as a key risk factor contributing to high rates of mortality in Barnsley. Athersley North is a peri-urban neighbourhood within the St Helens Ward of Barnsley with a stable population of around 1800 households with low FV consumption and high deprivation levels.^16^

There is a shopping area within Athersley with multiple fast food and processed food outlets and a local FV shop, and regular buses to Barnsley town centre (3 miles away), where a large local market has seven fresh FV stalls.

The study used Rapid Ethnographic Assessment.^17^ This included opportunistic conversations with local residents and key informants at local council health events in working men’s club, libraries and community centres; and information on the number of vouchers issued and reimbursed to retailers. All those contacted were informed about the research and verbal consent obtained. The study obtained approval from the Research Ethics Committee at the University of Sheffield Reference: 016340 [approved 18 October 2017].

### Developing the intervention and data collection methods

Four streets in Athersley North were chosen at random by residents attending the local community centre cafe. These four streets had 99 houses, of which 97 were occupied. All households were invited to contribute to the design of the voucher scheme at an ‘Information sharing and deciding’ workshop held with local stakeholders (24 January 2018). This meeting was attended by 14 stakeholder representatives: Alexandra Rose, Barnsley Metropolitan Borough Council Public Health team, elected councillors for the St Helen’s Ward, Community Shop, Be Well Barnsley (weight management service), My Best Life Barnsley (social prescribing project).

Following a discussion of the options available it was agreed that vouchers worth £5 per week would be offered to households (not individuals), and vouchers would be product specific (fresh FV) and redeemable only with local fresh FV market stalls, and FV shops.

### The intervention

**Fig. 1:**
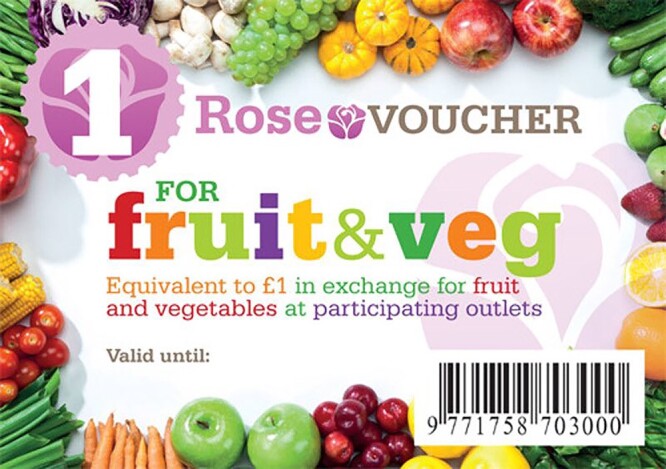

Every week five £1 vouchers were delivered to houses in a distinctive bright green envelope. Each £1 paper ‘Rose Voucher’ had attractive coloured pictures of fresh FV and a barcode and serial number (enabling vouchers to be linked to households) (Fig. 1). The envelope also included a letter with a simple, healthy vegetable or fruit-based recipe (Appendix 1), brief nutritional information that related to the recipe, and the following healthy eating message:

‘The **more** fruit and vegetables we eat (**especially veg**), the **healthier** we are and the **longer** we **live**. **5** portions of fresh fruit and vegetables a day is good, **7** is even better!’

Key features of this place-based household-level subsidy approach are as follows:

(a) vouchers are offered to households (not individuals),
(b) all households are eligible, regardless of size, type or income,
(c) households are encouraged to share vouchers, and
(d) vouchers are only redeemable with independent locally run outlets which only (or mostly) sell fresh FV. Vouchers are not redeemable at supermarkets.

In this initial field test, the vouchers were redeemable at six Barnsley town centre market FV stalls which only sold fresh FV, and the local FV shop which sold fresh FV and also eggs, nuts and plants. Rose Voucher posters were put up in the shop and stalls so that people knew that the vouchers would be accepted. Vouchers were reimbursed within 48 hours by the Alexander Rose Charity.^17^

### Implementing the intervention

The research team visited every household in order to explain the scheme and invite the household to join. Where there was no answer, a flyer about the scheme was left.

### Data collection

Researchers recorded conversations and observations with householders and shop and stall staff in field notes at each visit. Initially, joining the scheme was conditional on one adult from each household completing a pre-existing general health questionnaire.^19^ This six-page questionnaire asked questions about health and health-related behaviours including diet and requested personal identifiable information. However, only 10% of those approached returned a completed general health questionnaire. This was most likely due to worries that supplying personal information on health, mobility and income might have a negative impact on claims for state benefit.

As a result of the low completion rate this questionnaire was withdrawn and instead joining the scheme became conditional on householders answering four verbal ‘eating habits’ questions: ‘What did you have for your main meal yesterday?’, ‘Did you eat this meal alone or with others?’, ‘How often do you eat fruit?’ and ‘How often do you eat vegetables?’. Householders were also asked to provide verbal details of who lived in their house (see Table 1). These questions were repeated towards the end of the scheme in 10 months.

**Table 1:** Profile of participating households

	*Total sample N = 80 (100%)*
Single person	14 (17.5%)
Couple	21 (26.2%)
Parent + adult child	5 (6.2%)
Two parents + adult child(ren)	6 (7.5%)
Two parent family (children under 18)	21 (26.2%)
Single-parent family (children under 18)	13 (16.2%)

NB percentages may not sum to 100 due to rounding*.*

A total of 80 households joined the FRESH Street voucher scheme. These households included 141 adults and 63 children under 18 years (Table 1). The number of occupants per household ranged from 1 to 8. There were 32 two-person households (41%), 34 households (42.5%) included children under 18, one-third of which were single-parent families.

The research team regularly visited the shop and market stalls regularly in order to reaffirm that the vouchers should only be exchanged for fresh FV and obtain feedback on the impact of the scheme on trade. We recorded the unique ID numbers of the vouchers sent to individual households, details of the retail outlet where the voucher had been redeemed and the claim date.

### Data analysis

The distribution and redemption voucher logs provided details of the weekly spending patterns of each household—how many vouchers were spent, and when and where.

Informing households about the scheme, signing up households and delivering the weekly envelopes provided multiple opportunities for informal conversations. Our researchers prompted householders to share their views on the scheme using open questions. These informal opportunities enabled our researchers to have one or more conversations with 64 of the 80 households who joined the scheme. Field notes of these conversations were analysed with qualitative data analysis software^20^ and thematically analysed using themes derived from our theory about how the intervention might work: impact on FV consumption, food preparation patterns, new purchasing patterns, social interactions and longer term impact. During analysis three further themes emerged (thinking, prompting and not wasting). Excerpts in italics are quotes from field notes.

## Results

Initially, many householders were wary of joining the scheme. However, as positive news about the scheme spread more households joined. By six months into the scheme, 80 of the 97 (83%) eligible households had joined. As the scheme was well received by local stakeholders including local elected councillors, the local area council provided additional funding which enabled the scheme to be extended by a further 6 months.^21^

‘It’s a fantastic scheme which has had very good results - people are using it and the health benefits speak for themselves. I’d love to see it rolled out across the area council’s wards’ (Councillor Dave Leech, Chairman of the local area council)

At the end of the scheme, just four households had withdrawn (two never used the vouchers and two moved out of area). The majority (89.3%, 17 849/19 982) of vouchers issued were redeemed. Most (69.4%, 12 379/17 849) were used at the local FV shop and (30.6%, 5470/17 849) were redeemed at the FV stalls.

### Impact on FV consumption

There was no change in the proportion of householders who reported eating vegetables once a day or more (52%), but proportion of householders who reported eating fruit once a day or more increased from 60% at baseline to 76% at 10 months,

Eat more ‘because it's there all the time’ (Female, age 60)

Parents often commented on the impact on their children.

Without vouchers would have to make sure fruit lasted, with vouchers they can eat as much as they want. (Female, age not known)

Some householders reported that the vouchers subsidized (partially or totally) their pre-scheme spending on FV.

With vouchers she is saving money and eating a bit more veg – ‘a bit of both’ (Female, age 47)

For others, the vouchers enabled them to maintain spending on FV when budgets were stretched.

If she gets an unexpected bill and can't afford to do a big shop at least she can still get FV. (Female, age 26)

### Food preparation patterns

There was little change in response to the ‘eating habits’ main meal question. Most householders reported eating home-cooked meals, mostly ‘meat/fish and two veg’ type meals, or dishes cooked from multiple ingredients (e.g. stew, cottage pie, corned beef hash). Some reported using ready-made ingredients, (e.g. jar of pasta sauce) or convenience foods (e.g. ready-meal) or eating meals that required no cooking (e.g. pot-noodle, chicken salad and sandwiches). Although some commented that work often prevented them from eating together, most reported having eaten their main meal at home with other household members. However, some householders reported cooking from scratch more.

Used to cheat a lot at cooking (e.g. convenience/takeaway) - less so now. It helps that there is always FV in fridge. - cooking more than used to. (Male, 34)

Many mentioned using, sharing and saving the recipes provided with the vouchers.

Likes looking at the recipes, makes it more than just about vouchers. (Female, age 29)

### Thinking, prompting and not wasting

Many householders mentioned that the vouchers made them think about what they were eating, and prompted them to buy and eat more FV.

Vouchers make you think - reminder to get/have FV (Female, age 31)

Because it's free they get veg and because the veg is there they eat more. (Male, age 36)

Some said they felt they had to spend the vouchers as they did not want to waste them.

### Impact on health

Many householders, unprompted, mentioned the impact of the vouchers on their health.

Vouchers got her thinking about the importance of eating healthily, so she decided to join Slimming World and lost over 2.5 stone (Female, age 62)

### New purchasing patterns

Over half reported trying a greater variety of FV and/or trying new types of FV. The FV shop and stalls reported that the vouchers were bringing in new customers and that existing customers were spending more and buying a wider variety of FV. Householders reported that the local shop was convenient and better quality/fresher than the supermarket, but that the market stalls were cheapest. Some made the trip to the 3-mile trip to the market purely to spend their vouchers. Others did other shopping in the markets at the same time.

### Social interactions

There were indications that the scheme generated social interactions. Local children playing together in the streets often helped deliver the vouchers. Vouchers were swopped between households.

His sister took 2 [vouchers] last week and said she would make him some frozen meals. (Male, age 29)

### Longer term impact of the scheme

Nine months into the scheme, householders were asked what they would do when the scheme ended. Some said they would have to cut back, buy cheaper food, or less food, but many said they would continue to buy the same amount of FV.

Will carry on buying the same amount of veg - used to getting vouchers and have got into routine of going to get veg.

## Discussion

### Main finding of this study

The intervention combined a weekly subsidy scheme for fresh FV (5 × £1), with a vegetable- or fruit-based recipe, related brief nutritional information and healthy eating messages. This initial feasibility test found high levels of acceptance of the intervention by households offered the intervention, and strong support from local councillors and public health teams in the wider area.

Many householders reported that the vouchers made them think about what they were eating, and prompted them to buy and eat more FV. Many also reported new purchasing patterns.

### What is already known on this topic

Overall population adherence to dietary recommendations is sub-optimal.^8^ Diet-related ill health is socially patterned and a major contributor to health inequalities, e.g. obesity is most prevalent in lower income groups and those with lowest educational attainment.^22^ In the UK food costs limit the adoption of dietary recommendations^23^ for lower income households, where an estimated 42% of after-housing disposable income has to be spent to meet the Eatwell Guide recommendations.

Many populations at highest risk of COVID-19 have sub-optimal FV consumption, and are thus deficient in the many micro and phytonutrients which play a key role in supporting the immune system. Thus increasing FV consumption will help reduce susceptibility to COVID-19.^24^

In the UK, uptake of the national ‘Healthy Start’ programme is declining rapidly with just 48.0% of those eligible in England in the programme.^14^ Healthy Start vouchers are spent in supermarkets in low-income areas which tend to offer fewer fruits and vegetables compared to more wealthy areas^2^ and most ‘Healthy Start’ vouchers for children under 1 year are redeemed for infant formula rather than FV.^25^ It is feasible to offer vouchers for FV from FV market stalls to vulnerable families in receipt of Healthy Start vouchers,^18^ but offering local supplier-specific fresh FV vouchers to every household in geographically defined communities has not been previously studied in the UK.

### What this study adds

We successfully developed and feasibility tested a place-based household-level subsidy approach to improving diet, health and the wider environment in one geographically defined deprived community. This provides the first information as to what the key features of such an intervention might be.

This novel approach sought to influence household’s choices on four levels^26^: ‘providing information’ (on healthy eating, nutrition and health behaviour change), ‘enabling choice’ (providing vouchers), ‘guiding choice by incentives’ (providing vouchers), and ‘altering the local food retail environment’ *(*by creating a steady demand, which in turn helps vendors consistently offer items that were previously not profitable). This area-based programme was been designed to reduce food insecurity, increase daily consumption of fresh FV, and improve dietary quality by supporting healthy dietary habits, and increasing exposure to healthy food prompts.

As all households were eligible regardless of size, type or income, everyone could access the vouchers without having to demonstrate need or be referred. This helped avoid the stigma associated with schemes which target individuals (such as Healthy Start), and focused the impact on one community where many people were experiencing food insecurity, and consuming diets high in energy-dense, nutrient poor, processed foods and low in FV.

Vouchers were only redeemable with independent locally run outlets selling fresh FV (not supermarkets). This minimized the likelihood of the vouchers being exchanged for items other than fresh FV and also supported local markets and traders.

### Limitations of this study

We tested the feasibility of a local, temporary, small-scale scheme over a 12-month period. Information on the impact of the scheme was limited to self-reports by householders to the research team. These self-reports were vulnerable to both responder and social acceptability bias. We were unable to assess how much or little the vouchers increased FV consumption and expenditure or subsidized existing expenditure. Further research is required to rigorously assess the impact of the scheme, ideally over a much longer time period.

## Conclusion

This study demonstrated the broad acceptability of a place-based, household-level weekly subsidy scheme in an area of high deprivation and low FV consumption. Households who joined the scheme reported that the vouchers made them think about what they were eating, and prompted them to buy and eat more FV. Many also reported new purchasing patterns.

Further research is required to measure the impact of this intervention on the wide range of determinants of health for individuals, households, areas and local economies and assess the value of this approach to creating sustainable and healthy diets, eating behaviours and food systems.

## Notes

**Clare Relton,** Senior Lecturer

**Mark Strong,** Professor

**Megan Blake,** Senior Lecturer

**Mary Crowder,** Research Associate

## Appendix 1. Weekly recipes

Week 1: no recipe.

Week 2: no recipe.

Week 3: Clare’s Carrot and Lentil Soup.

Week 4: Roasted cauliflower cheese.

Week 5: 10 minute cabbage and bacon.

Week 6: Chickpea and spinach stew.

Week 7: Leek and nettle soup.

Week 8: Tomato and onion pasta and green salad.

Week 9: Broccoli, onion and potato soup.

Week 10: Homity pie.

Week 11: Courgette and tomato frittata.

Week 12: Vegetable risotto.

Week 13: Steamed veg with herby dressing.

Week 14: Hummus and crunchy veg.

Week 15: One day detox.

Week 16: Clare’s summer protein.

Week 17: Greek-style roast fish.

Week 18: Courgette soup.

Week 19: The green smoothie formula.

Week 20: Fruity summer Charlotte.

Week 21: Garlicky green beans.

Week 22: Courgette fritters.

Week 23: Classic carrot cake.

Week 24: Easy vegetable stir-fry.

Week 25: Roasted vegetables.

Week 30: Seasonal vegetables with handy tips (incl bubble & squeak, vegetable pasta bake; vegetable pie).

Week 31: Fruit and vegetable portion guide.

Week 32: Leek, potato & bacon bake.

Week 33: Cranberry cinnamon poached pears.

Week 34: Stir-fried curly kale with chilli and garlic.

Week 35: Vegetable stew.

Week 36: Leek and potato soup.

Week 37: Chicken and vegetable soup.

Week 38: Spicy root and lentil casserole.

Week 39: Mixed vegetable and bean soup.

Week 40/41: Brussels sprouts with bacon and chestnuts.

Week 42: Aromatic carrot and parsnip soup.

Week 43: Butternut squash soup.

Week 44: Carrot and butterbean soup.

Week 45: Banana, nut and molasses flapjack.

Week 46: Mushroom soup.

Week 47: Banana ice cream.

Week 48: Sweet potato fried rice.

Week 49: Savoy cabbage with almonds.

Week 50-52: Slow-cooked root vegetable soup.
